# Evaluation of Chemical Composition, Radical Scavenging and Antitumor Activities of *Satureja hortensis* L. Herb Extracts

**DOI:** 10.3390/antiox10010053

**Published:** 2021-01-05

**Authors:** Kristina Bimbiraitė-Survilienė, Mantas Stankevičius, Simona Šuštauskaitė, Agnieszka Gęgotek, Audrius Maruška, Elżbieta Skrzydlewska, Zita Barsteigienė, Ieva Akuņeca, Ona Ragažinskienė, Audronis Lukošius

**Affiliations:** 1 Instrumental Analysis Open Access Center, Faculty of Natural Sciences, Vytautas Magnus University, Vileikos 8, LT-44404 Kaunas, Lithuania; kristina.bimbiraite-surviliene@vdu.lt (K.B.-S.); mantas.stankevicius@vdu.lt (M.S.); ieva.akuneca@inbox.lv (I.A.); 2 Department of Pharmacognosy, Medical Academy of Lithuanian University of Health Sciences, LT-44307 Kaunas, Lithuania; gabrieletta@gmail.com (S.Š); zita.barsteigiene@lsmuni.lt (Z.B.); audronis.lukosius@lsmuni.lt (A.L.); 3 Department of Analytical Chemistry, Medical University of Bialystok, PL-15-230 Bialystok, Poland; agnieszka.gegotek@umb.edu.pl (A.G.); elzbieta.skrzydlewska@umb.edu.pl (E.S.); 4 Botanical Garden of Vytautas Magnus University, Vytautas Magnus University, LT-46324 Kaunas, Lithuania; ona.ragazinskiene@vdu.lt

**Keywords:** summer savory, radical scavenging activity, essential oil, phenolic compounds, melanoma cells, melanocytes

## Abstract

*Satureja hortensis* L. is an annual herbaceous plant of the Lamiaceae Lindl. family. *S. hortensis* L., related to thyme and rosemary, is used as spice and traditional medicinal herb in Europe. Mainly due to the polyphenols contained in *S. hortensis* L., this plant exhibits multiple biological effects. However, therapeutic effects on cells, including skin tumors, have not yet been studied. Therefore, the aim of this study was to compare the composition and the resulting antioxidant as well as biological properties [on melanocytes and melanoma cells] of summer, savory growing in botanical garden of Vytautas Magnus University in middle Lithuania climatic conditions, collected during various phases of vegetation. It has been shown that the budding phase alcohol extract of this plant contains the largest amounts of polyphenols, including rutin and rosemary acid, which promote the radical scavenging activity and antioxidant properties. In contrast, the extract from the massive flowering phase already at a concentration of 12.5 µg/mL reduces the survival of melanoma cells to 60% with 90% melanocytes survival. In addition, extracts from beginning of flowering and end of flowering at a concentration of 25 µg/mL, containing significantly less rutin and rosmarinic acid, in combination with irradiation of cells with UVB, significantly increased the lipid peroxidation process, particularly in melanoma cells. These data indicate the possibility of using extracts from *S. hortensis* L. to modulate/differentiate the metabolism of normal and tumor skin cells.

## 1. Introduction

Healing and aromatic herbs, the diversity of their species, reproduction, and issues of enhancing human health are analyzed based on the Global Plant Protection Strategy and the Convention on Biological Diversity [1,2]. Medicinal and culinary plants, which are characterized by a high content of essential oils, are the subject of food chemistry research due to growing concerns about the toxicity of synthetic food additives and the usefulness of conventional preservatives used in everyday food. Medicinal plants also usually contain relatively large amounts of compounds with antioxidant properties. Therefore, they are beneficial to human health and, therefore, can be used as natural preservatives and dietary supplements. They can also be used to support pharmacotherapy, especially cancer therapy [3,4].

*Satureja hortensis* L. is an annual herbaceous plant of Lamiaceae Lindl. Family. *S. hortensis* L., related to thyme and rosemary, is native to North Africa, Middle East, and Central Asia, Southern and Southeastern Europe. Satureja species contain, in various concentrations, many phenolic and volatile components such as carvacrol, γ-terpinene, thymol, p-cymene, β-arylene, linalool, and other terpenoids [5]. The mentioned compounds are synthesized mainly in plants in order to protect them against harmful environmental factors, including UV radiation contained in the sunlight. However, the exact chemical composition depends on the Satureja species, as well as the location and the prevailing climate [6]. In Asia it is widely cultivated as a culinary herb. Due to the attractive aroma, savory herbs, both dried and fermented, are used as flavoring substances and natural preservatives in food processing [7]. This plant is used as a traditional medicinal herb in Europe, and the medicinal plant raw material of savory is the aerial part of the plant—*Herba Satureae* [8,9]. This plant contains two main groups of biologically active compounds, namely phenolic compounds and essential oils [10,11,12,13,14]. Phenolic compounds exert multiple biological effects such as antioxidant, free radical scavenging and anti-inflammatory properties [15]. Due to accumulated compounds, *S. hortensis* L. possess antioxidant, antibacterial, antispasmodic, antidiarrheic, hypoglycemic, and anti-hypercholesterolemic effects [16,17,18,19,20,21]. However, environmental factors strongly influence the biosynthesis of polyphenols known as secondary metabolites of medicinal and aromatic plants, which means that the exact therapeutic activity of *S. hortensis* L. depends on the place of origin [22].

Redox homeostasis of human body cells, including skin cells, is important for intracellular metabolism, which determines the proper functioning of the organism. The redox balance depends on the amount of free radicals produced and the cell’s antioxidant capacity to counteract their cellular activity [23]. However, as a result of disorders of cell metabolism, as well as the action of environmental factors such as UV radiation, there is a disruption of cell redox balance and the development of disease states, including cancer [24]. This situation is usually accompanied by overproduction of highly reactive oxygen species (ROS), which act as short-term signaling molecules and stimulate the production of additional mediators, including lipid peroxidation products [25]. Lipid peroxidation products play an important causal role in the initiation and development of diseases [26]. Skin epidermis subtracts unique mixture of lipids (mainly ceramide, cholesterol, and other fatty acids) that forms natural permeability barrier. Free radicals such as superoxide radicals, hydroxyl radicals, and singled oxygen disrupts the natural defense of the skin by reacting with skin lipids, which leaves skin exposed to negative environment. Antioxidants such as polyphenolic compounds scavenge free radicals by inhibiting the initiation or propagation of oxidative chain reactions and can protect organism against serious diseases including cancer [27]. Essential oils of aromatic medical plants are the source of the bioactive compounds that may protect skin cells due to their anti-inflammatory, antibacterial and antioxidant properties [28].

Therefore, the aim of the study was to assess the composition and the resulting antioxidant properties of summer savory (*Satureja hortensis* L.) growing in Kaunas Botanical garden of Vytautas Magnus University in Middle Lithuania climatic conditions, collected during various phases of vegetation. The research included chemical analysis of the aerial parts of summer savory and the ability to capture free radicals by *S. hortensis* L. extracts using separation methods. In addition, the ability of these extracts to counteract lipid peroxidation in human epidermal cells [melanocytes and melanoma] was evaluated under physiological conditions and after induction of oxidative stress by UV irradiation of the cells.

## 2. Materials and Methods

### 2.1. Chemicals

For spectrophotometric analysis acetonitrile (99.9%) and Folin–Ciocalteu reagent were purchased from Merck, Darmstadt, Germany, methanol (99.9%) and trifluoroacetic acid (98%) were from Sigma-Aldrich, Saint Louis, MO, USA; acetic acid (99%) was from Lachema, Neratovice, Czech Republic, aluminum chloride was from Chempur, Piekary Sląskie, Poland; sodium carbonate and hexamethylenetetramine (C_6_H_12_N_2_) (99.9%) were from Roth, Karlsruhe, Germany; 2.2-diphenyl-1-picrylhydrazyl (DPPH) was from Sigma, Germany. Standards for HPLC qualitative analysis: *trans*-synapic acid, rosmarinic acid, and hesperetin were purchased from Fluka, Steinheim, Germany, *trans*-p-coumaric acid was from Chromadex, Irvine, CA, USA and rutin was from Sigma, Steinheim, Germany. Bidistilled water was prepared using distillation system Firstreem^TM^ Cyclon^TM^, Loughborough, UK.

### 2.2. Raw Material

Areal parts of the summer savory (*Satureja hortensis* L.) were harvested at 5 different vegetation phases from ex situ collection of Medicinal and aromatic plants sector at Kaunas Botanical Garden of Vytautas Magnus University in Lithuania (map coordinates: 54.870453, 23.908354) in 2014. Vegetation phases and dates of collection are following intensive growth (25 June 2014), budding (21 July 2014), floral budding (24 July 2014), full flowering (28 July 2014) and end of flowering (20 August 2014). Harvested areal herbal parts were spread in a thin layer to dry in a well-ventilated room protected from direct sunlight. The dried herbal raw material was stored in textile bags at ambient temperature. Air-dried raw material was used for further research.

### 2.3. Extraction Using Organic Solvents

For preparation of methanolic extracts 0.5 g of dry plant material was extracted in 20 mL methanol—water solution (75:25 (vol.)) for 24 h shaking at the rate of 250 rpm. Extracts were filtered using paper filter and later 0.45 μm pore size PVDF membrane filter and stored in the refrigerator (4 °C).

### 2.4. Volatile Compounds Determination by GCMS

Volatile compounds of summer savory (*Satureja hortensis* L.) samples were extracted using solid-phase microextraction (SPME). The SPME was performed in headspace of the sample in 10 mL glass bottles closed with screw caps with silica/Teflon septa using Stableflex fiber coated with 50/30 μm DVB/PDMS layer (Supelco, Bellefonte, PA, USA). 0.025 g of herbal raw material samples were weighed for the extraction. Headspace extraction of the essential oil was carried out at 30 °C temperature for 10 min. The thermal desorption was carried out at 280 °C for 1 min. Automated SPME system was coupled with the chromatographic module. For determination of repeatability of results, extractions were repeated three times (*n* = 3).

GC-2010 gas chromatograph and GCMS-QP2010 mass spectrometer (Shimadzu, Kyoto, Japan) were used for the determination of essential oil components. An automated injector AOC-5000 (Shimadzu, Kyoto, Japan), with SPME system was used for sample preparation and injection. The RTX-5MS column (Restek, Bellefonte, PA, USA) 30 m length, 0.25 mm internal diameter and 0.25 μm stationary phase thickness, was used for separation of volatile compounds. For mass spectrometry an electron ionization detector set at 70 eV was used. The conditions of GC-MS method were as described before [29,30]. Initial column temperature 60 °C, injector temperature 280 °C, ion source temperature 260 °C, interface temperature 260 °C. The injection was performed at split ratio 1:10. The temperature was increased gradually from 60 °C to 150 °C at 5 °C/min and from 150 °C to 280 °C at 20 °C/min and kept for 3 min. Total analysis time was 30.5 min. Compounds were determined analyzing mass spectra and comparing to the electronic NIST v14.1 library of mass spectra.

### 2.5. Phenolic Compounds Determination

Total phenolic and flavonoid contents as well as free radical-scavenging assay were determined using UV-VIS spectrophotometer (Spectronic 1201, Milton Roy, Houston, TX, USA). All experiments were performed using 1 cm optical path length quartz cuvette. In order to harmonize results, all spectrophotometric analysis results were calculated in rutin equivalents (mg of rutin standard per 1 g of dry raw material: RE mg/g). Procedures were as previously described by Stankevičius et al., 2010 [31].

Total phenolic content was determined using the Folin–Ciocalteu colorimetric method. An aqueous stock solution of 4% sodium carbonate (Na_2_CO_3_) was prepared and stored in refrigerator (4 °C). 100 μL of sample extract was added to 3000 μL of stock solution, mixed, and 100 μL 2 N Folin–Ciocalteu reagent was added. After 30 min incubating at room temperature the sample absorbance was measured at 760 nm. Results were calculated in rutin equivalents (RE mg/g).

The separation and identification of phenolic compounds and determination of their radical scavenging activity were carried out using modular HPLC system consisted of mobile phase binary pump (Hewlett Packard, Waldbronn, Germany), autoinjector (Perkin Elmer, Norwalk, CT, USA), chromatographic column (125 mm × 4.60 mm LiChrospher 100 RP-18e, particle size 5 μm (Merck, Darmstadt, Germany), UV detector (λ = 254 nm) “Spectra 200” (Spectra Physics, Norwalk, CT, USA), DPPH reagent pump (Phoenix 20 CU, Carlo Erba Instruments, Milan, Italy), DPPH reaction detector (λ = 517 nm) “Linear UVIS 200” (Linear Instruments, Reno, NV, USA) and data processing system. Mobile phases were as follows: A—bidistilled water, acidified with trifluoroacetic acetic acid (0.05% vol.), B—methanol. Following mobile phase gradient was applied: from 0 min. to 4 min. component B concentration is changed from 1% to 30%, on 20 min. B is set to 43%; on 30 min. B is set 50%, on 40 min.—B is set to 99% and hold for 5 min. Flow rate: 0.75 mL/min, injection volume 10 µL. Compounds were determined according to the retention times using external standard method. Retention time of standard compounds was accordingly: *trans*-synapic acid—19.44 min., *trans*-p-coumaric acid—23.27 min., hesperetin—23.83 min., rutin—24.73 min., rosmarinic acid—25.84 min.

The content of total flavonoids was determined by AlCl_3_ colorimetric method. A stock solution containing 60 mL of methanol (100%), 3 mL of acetic acid (33.0%), 12 mL of hexamethylenetetramine (5%), 9 mL of aluminum chloride (10%) and 60 mL of bidistilled water was prepared and stored in the refrigerator (4 °C). Total flavonoid content was determined mixing 80 μL of extract with 1920 μL of stock solution. After 30 min incubation at 4 °C the absorbance was measured at 407 nm. Results were calculated in rutin equivalents (RE mg/g).

### 2.6. Radical Scavenging Activity Measurement

Radical scavenging activity of *S. hortensis* L. extracts was assessed using DPPH radical scavenging method [31]. DPPH solution was prepared by dissolving 4 mg of DPPH in 50 mL of acetonitrile, 50 mL of methanol and 100 mL of prepared acetate buffer (1.36 g of sodium acetate dissolved in 100 mL of bi-distilled water, pH 5.5 was adjusted using 33% aqueous acetic acid). 77 μL of sample extract was added to 3000 μL of radical solution, mixed by shaking vial and after 15 min. incubation time the absorbance was measured spectrophotometrically at the wavelength of 515 nm. The prepared solution was kept in darkness. The measurements were performed triplicate. Results were expressed in rutin equivalents (RE mg/g).

### 2.7. Biological Effects of S. hortensis L.

Human skin melanocytes (ATCC-PCS-200-012) and melanoma cells (ATCC-HTB-72, designation SK-MEL-28) used in experiment were obtained from American Type Culture Collection and cultured in a humidified atmosphere of 5% CO_2_ at 37 °C. Human melanocytes were cultured in medium 254, while melanoma cells were cultured in Eagle’s Minimum Essential Medium (EMEM). All media were supplemented with 10% fetal bovine serum (FBS) and antibiotics (50 U/mL penicillin and 50 μg/mL streptomycin). All sterile reagents for cell culture were obtained from Gibco (Grand Island, NY, USA).

When the cells reached 70% confluence, they were exposed to UV radiation using Bio-Link Crosslinker BLX 312/365; Vilber Lourmat, Germany. UVB radiation dose was 300 mJ/cm^2^ and was chosen corresponding to 70% cell viability measured by the methyl tetrazolium (MTT) assay [32] described in detail below. The distance between cells and lamps was 15 cm, what gave power density at 4.08 mW/cm^2^ for UVB (312 nm). Control cells were incubated in parallel without irradiation.

To examine the effect of *S. hortensis* L. extracts, skin cells were incubated for 24 h with extracts for 24 h under standard conditions in medium containing 12.5, 50, 75, and 100 µg/mL plant extract in 72% ethanol or were irradiated with UVB and then treated for 24 h with extract as above. The concentration of *S. hortensis* L. extracts was chosen to correspond to 100% cell viability compared to control cells measured in the MTT assay [32]. The test uses the ability of active enzymes present in living and metabolically active cells to reducing the tetrazolium dye MTT 3-(4,5-dimethylthiazol-2-yl)-2,5-diphenyltetrazolium bromide to its insoluble formazan. For this test, cells were seeded into a 96 well plate in 200 μL of growth media at 5 × 10^3^ cells/well and after 24 h of growing subjected to UVB radiation or plant extracts. Following 24 h incubation culture medium was removed and MTT solution in PBS (0.25 mg/mL) was added to each well. The plate was incubated for 1 h at 37 °C and 5% CO_2_. All medium was removed from each well and 200 μL of DMSO was added to cells lysis. The level of formazan formed during incubation was read at 570 nm on a Multiskan GO Microplate Spectrophotometer (Thermo Fisher Scientific, Waltham, MA, USA) and calculated as a percentage of results obtained for control sample.

### 2.8. S. hortensis L. Effect on Cells Viability

Cells viability following treatment with extracts was measured using MTT assay [32]. The test uses the ability of active enzymes in functioning cells to reducing the tetrazolium dye MTT 3-(4,5-dimethylthiazol-2-yl)-2,5-diphenyltetrazolium bromide to its insoluble formazan that was measured spectrophotometrically at 570 nm.

### 2.9. Effect of S. hortensis L. on Lipid Peroxidation

Lipid peroxidation was estimated by measuring the level of malondialdehyde (MDA) by GC/MS in selected ion monitoring (SIM) mode, as the *O*-pentafluorobenzyl-oxime (*O*-PFB-oxime) or *O*-pentafluorobenzyl-oxime-trimetylsilane (*O*-PFB-oxime-TMS) derivatives using benzaldehyde-D6 as an internal standard [33]. MDA was analyzed using a 7890A GC—7000 quadrupole MS/MS (Agilent Technologies, Palo Alto, CA, USA) equipped with a HP-5MSms capillary column (30 m length, 0.25 mm internal diameter, 0.25 µm film thickness). The ions used were *m*/*z* 333.0 and 181.0 for MDA-PFB-TMS and *m*/*z* 307.0 for IS derivative. Obtained results were normalized for milligrams of protein.

### 2.10. Statistical Data Analysis

The experimental data were processed by MS Excel 2003 (Microsoft, Redmond, WA, USA) and Origin 8.0 (Northampton, MA, USA) software. All the assays were performed in triplicate (*n* = 3) and the results were calculated as mean value ± standard deviation. The Shapiro-Wilk and the Leven tests were used to check the normality of the data distribution and the homogeneity of variance. Pearson’s correlation coefficient (*r*^2^) was chosen for the correlation signal strength description. Student’s *t*-Test and F-Test were used to compare means of results during all vegetation phases versus V1 intensive growth phase. Rutin standard solutions were used to build up the calibration graphs.

## 3. Results

**Figure 1:**
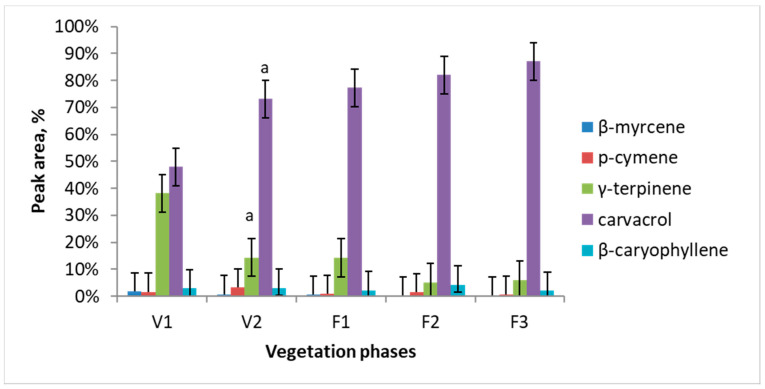
Dynamics of essential oil major components of *Satureja hortensis* L. during the vegetation determined using gas chromatography—mass spectrometry. Vegetation phases: V1—intensive growth, V2—budding, F1—beginning of flowering, F2—massive flowering, F3—end of flowering. Mean values ± SD from three independent experiments are presented. ^a^ statistically significant differences versus V1, (*p* < 0.05).

Major essential oil components of *Satureja hortensis* L. raw material were determined using SPME and gas chromatography-mass spectrometry technique. Totally 21 volatile compounds were identified and quantified. Results of this analysis revealed five dominating compounds, which are: β-myrcene, p-cymene, γ-terpinene, carvacrol, and β-caryophyllene. Essential oil quantitative composition and its dependence on the vegetation phase are presented in Figure 1. The major compound in all the vegetation phases was carvacrol. Carvacrol is predominant and its amount accumulates during all vegetation phases from 47% in V1 to 87% in F3. The maximum concentration of γ-terpinene observed during the intensive growth is later considerably decreased (circa 10 times) and is only 6% at the F2 and F3 phases.

**Figure 2:**
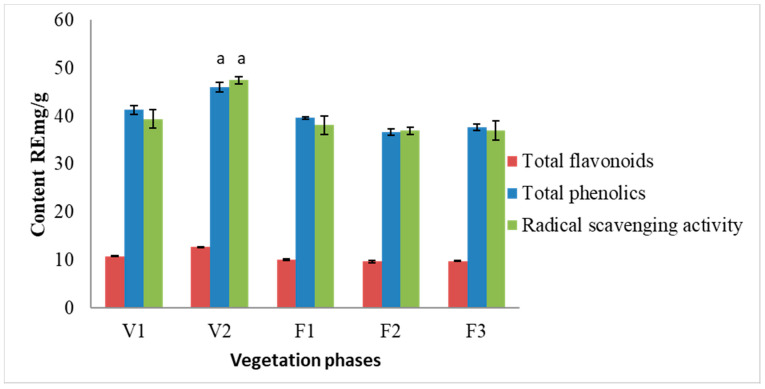
Variation of total flavonoid and phenolic compounds and radical scavenging activity of methanolic extracts of *S. hortensis* L. from different vegetation phases, determined using spectrophotometric methods. Mean values ± SD from three independent experiments are presented. ^a^ statistically significant differences vs. V1 phase, *p* < 0.05.

The results of the spectrophotometric analysis are presented in Figure 2. Total phenolic content did not change significantly during F1–F3 vegetation phases. The ratio between total phenolic content and total flavonoid content remained constant, ca. 1:4. The highest amounts of phenolic compounds (46.01 RE mg/g) and flavonoids (12.81 RE mg/g) were observed in *S. hortensis* L. raw material extracts at the V2 phase. The highest radical scavenging activity was determined in the samples of V2 phase (47.49 RE mg/g). The obtained results between total phenolic content and radical scavenging activity were statistically dependent on each other. The results from the present study indicate a strong correlation between the radical scavenging activity and the content of total phenolics (*r*^2^ = 0.798) as well as between radical scavenging activity and total flavonoid content (*r*^2^ = 0.894).

**Figure 3:**
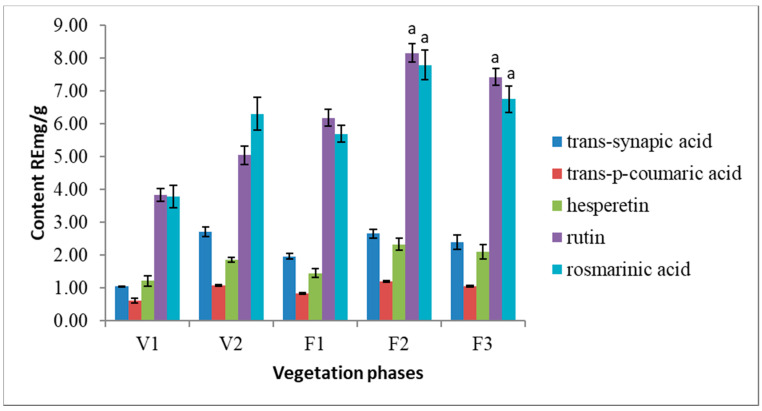
Variation of the main phenolic compounds in *Satureja hortensis* L. herb samples during vegetation phases determined using high-performance liquid chromatography—DPPH. V1—intensive growth, V2—budding, F1—beginning of flowering, F2—massive flowering, F3—end of flowering. Mean values ± SD from three independent experiments are presented. ^a^ statistically significant differences, (*p* < 0.05).

HPLC analysis revealed 5 phenolic compounds dominating in *S. hortensis* L. extracts. Following phenolic acids were identified in the extracts: *trans*-synapic acid, *trans*-p-coumaric acid, rosmarinic acid as well as flavonoids—rutin and hesperetin (Figure 3). Rutin and rosmarinic acid are the most abundant nonvolatile compounds in *Satureja hortensis* L. herbal raw material. The results of present research show that the amount of rosmarinic acid varies from 3.79 RE mg/g to 7.79 RE mg/g. The amounts of rutin is ranging from 3.83 RE mg/g to 8.16 RE mg/g in the air-dried raw material. In addition, a steady increase of rutin is observed from early V1 to late F2 vegetation phase. *Trans*-synapic acid, *trans*-p-coumaric acid, and hesperetin were determined at significantly lower quantities.

**Figure 4:**
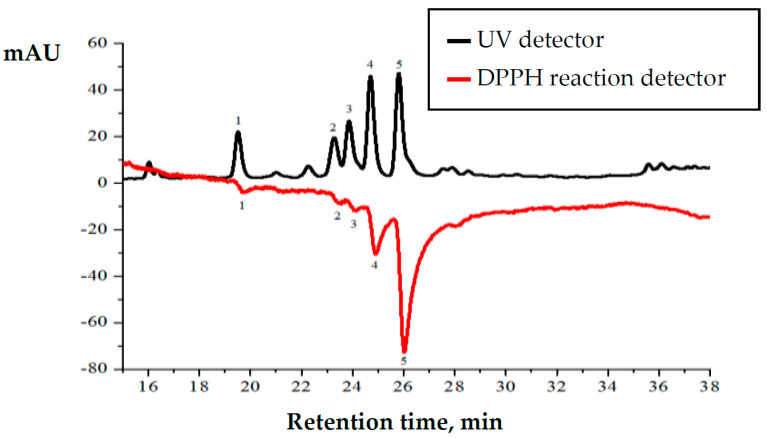
Chromatographic profile of *Satureja hortensis* L. raw material methanolic extract during the F2 phase. 1—*trans*-sinapic acid 19.44 min., 2—*trans*-p-coumaric acid 23.27 min., 3—hesperetin 23.83 min., 4—rutin 24.73 min., 5—rosmarinic acid 25.84 min.

Plant extracts of summer savory (*S. hortensis* L.) were analyzed using high-performance liquid chromatography coupled with DPPH radical scavenging detector (Figure 4). Chromatogram shows two signals plotted as mirror image. Upper chromatogram shows separated compounds detected using UV detector and negative chromatogram shows DPPH radical scavenging activity of the separated compounds [34].

HPLC analysis revealed that the highest amounts of identified compounds and their anti-radical activity are determined in the methanolic extracts of collected raw material at the F2 phase. Presented HPLC chromatogram (Figure 4) shows that rutin (peak number 4) and rosmarinic acid (peak number 5) are the predominant compounds. Although the quantities of identified compounds are similar, post-column DPPH radical scavenging profile shows that radical scavenging activity of rosmarinic acid is three times higher than that of rutin. Post-column DPPH assay revealed that radical scavenging activity of *trans*-synapic acid, *trans*-p-coumaric acid and hesperetin is significantly lower.

The strong correlation (*r*^2^ = 0.9759) between content of rosmarinic acid and total radical scavenging activity of the extract was determined earlier [34]. Rosmarinic acid is the strongest antioxidant in a mixture, and its contribution to the total radical scavenging activity is the highest. Correlation coefficients calculated for other compounds were lower, but they are also considered to be strong, as for example *trans*-synapic acid—*r*^2^ = 0.772, *trans*-p-coumaric acid *r*^2^ = 0.832, hesperetin *r*^2^ = 0.787.

The cytotoxicity of alcoholic extracts from *S. hortensis* L. was tested against human melanocytes and human malignant melanoma cells. Cells viability was assessed by their ability to reduce MTT to formazan dye after 24-h exposure of cells to *S. hortensis* L. extract at various concentrations ranging from 12.5 to 100 μg/mL. A concentration-dependent inhibitory effect of the extracts on the viability of melanocytes and melanoma cancer cells was observed. Cytotoxicity was considered whenever the percentage of cell survival was less than 70%. The IC50 for each of extracts were estimated as IC50_V1_ = 1.250 mg/mL, IC50_V2_ = 8.535 mg/mL, IC50_F1_ = 325 µg/mL, IC50_F2_ = 160 µg/mL, IC50_F3_ = 25 µg/mL in the case of melanocytes and IC50_V1_ = 285 µg/mL, IC50_V2_ = 270 µg/mL, IC50_F1_ = 195 µg/mL, IC50_F2_ = 45 µg/mL, IC50_F3_ = 140 µg/mL for melanoma cells.

**Figure 5:**
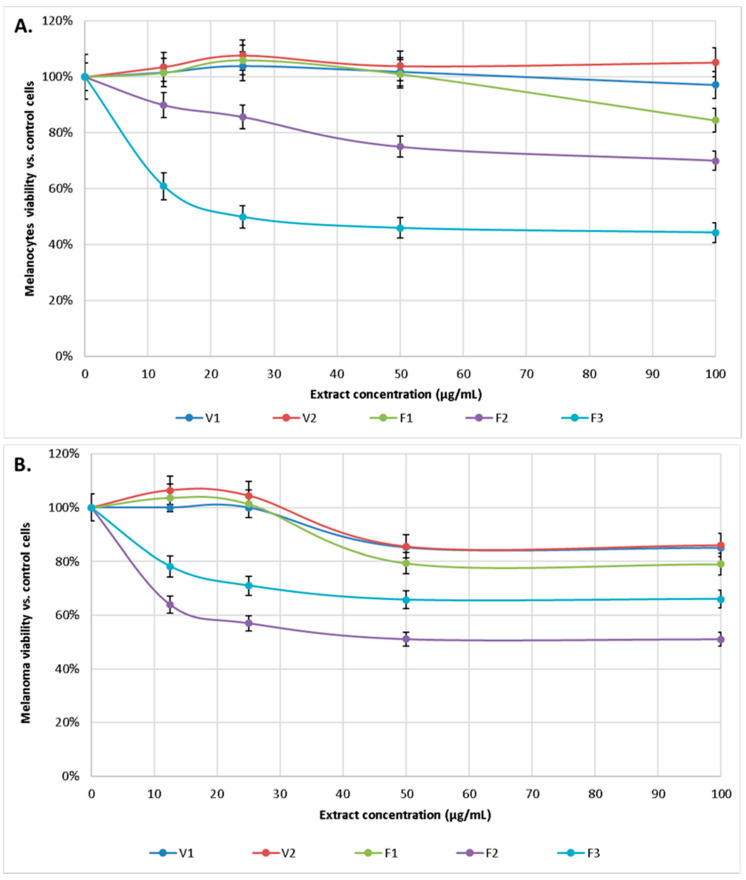
Effect of extract of *S. hortensis* L. (concentration range 12.5–100 µg/mL) on melanocytes (**A**) and melanoma (**B**) viability. Mean values ± SD of three independent experiments are presented.

Extracts V1, V2, and F1 at the concentrations used practically did not affect melanocytes survival, while extract F2 gradually reduced melanocytes survival to about 70% (for a concentration of 100 μg/mL). In contrast, extract F3 already at a concentration of 12.5 μg/mL reduced the survival of melanocytes to 60%, and at higher concentrations reduced it to about 50%. In contrast, V1, V2, and F1 extracts were also not toxic to melanoma cells, while F2 extract showed cytotoxicity from 50 μg/mL, and F3 from 12.5 μg/mL (Figure 5).

**Figure 6:**
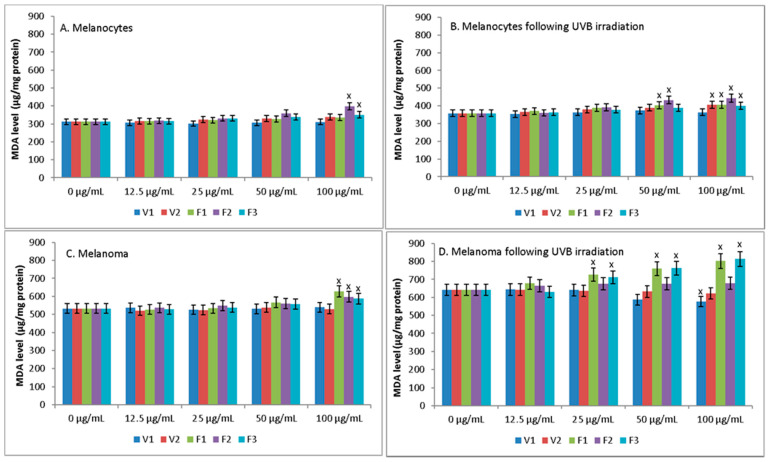
The level of MDA in melanocytes (**A**,**B**) and melanoma cells (**C**,**D**) following treatment with extract of *Satureja hortensis* L. (concentration range 12.5–100 µg/mL) and/or UVB (300 mJ/cm^2^) irradiation. Mean values ± SD of three independent experiments are presented. ^x^ statistically significant differences vs. control group (0 μg/mL), *p* < 0.05.

None of the *S. hortensis* L. extracts up to a concentration of 50 μg/mL increased lipid peroxidation, estimated by MDA, in both melanocytes and melanoma cells (Figure 6). Only at a concentration of 100 μg/mL extracts of phases F2 and F3 increased peroxidation in melanocytes and extracts of F1–F3 in melanoma cells. Whereas UVB radiation increased lipid peroxidation in both melanocytes and melanoma cells, additionally causing greater vulnerability of cells to extracts, which was especially manifested in the case of melanoma cells. While, in the case of UV irradiated melanocytes, statistically significant increase of MDA was observed only at the high concentration of some extracts (50 μg/mL of extracts F1-2 and 100 μg/mL of extracts V2 and F1–F3), for UV irradiated melanoma cells this increase was observed for extracts F1 and F3 already at a concentration of 25 μg/mL.

## 4. Discussion

Ingredients of essential oils/extracts from aromatic herbs and dietary/medicinal plants include, but are not limited to, phenols and terpenes—compounds that, alone or in synergy with others, can modulate metabolic processes in human body cells [35,36]. The action of these compounds based on antioxidative, anti-inflammatory, antimutagenic, and antiproliferative mechanisms, improves immune function and induces enzymes that intensify metabolic processes, including resistance to the harmful oxidative effects of external environment factors, such as UV radiation as well as the modulation of resistance to many drugs [37,38]. Therefore, natural oils/extracts, including *S. hortensis* L., may play an important role in the prevention and treatment of various diseases, including cancer.

UVB radiation used in this study was chosen as an example of a pro-oxidative environmental factor characterized by repetitive, strong and direct action. Additionally, this factor affecting every person regardless of age, work, place of residence, as well as it is also often used in phototherapy, in contrast to the UVA that requires the additional use of a psoralen sensitization. Also, it is not possible to select a universal radiation dose that would transpose the average exposure of human skin to the dose having the same effect in in vitro cultured skin cells. That was the reason for choosing only the UVB radiation in dose, causing significant changes in cell metabolism, especially in the field of redox homeostasis.

The results of this study show that terpenes predominate among volatile substances of *S. hortensis* L. extract, especially carvacrol. However, in the alcohol extract large amounts of phenolic compounds are observed, especially rutin and rosemary acid, which is visible, especially also in the V2 phase. The studies published by other researchers reveal different results, when *Satureja hortensis* L. raw material samples are collected from various natural sampling sites. Studies carried out by Gulluce et al. (2003) and Hashemi et al. (2012) demonstrated that thymol and γ-terpinene are the predominant components of essential oil. Carvacrol, p-cymene, β-myrcene, and α-terpinene were determined at lower concentrations [17,39]. Comparative studies conducted by Baker et al. (2004) indicated that essential oils obtained from *S. hortensis* L. cultivated in Turkey possess prevailing quantities of carvacrol. However, the samples collected from natural sampling sites possess predominant component-thymol [40]. Carvacrol and thymol are reported to be antioxidant compounds in essential oil of savory compounds that possess antioxidant activity [10].

Due to the high content of biologically active compounds in the *S. hortensis* L. extract, they can modify the metabolism of skin cells. The survival of skin cells, especially melanoma, was significantly reduced by extracts F2 and F3, which can be attributed to the relatively high concentrations of nonvolatile polyphenols rutin, rosmarinic acid, and the high level of volatile monoterpenoid phenol—carvacrol contained in the extracts. Literature data indicate that carvacrol, the dominant monoterpen also in *S. montana* L., very strongly inhibits cell growth in various cell models, particularly in the A549 tumor cell line [41]. In addition, a comparative assessment of the cytotoxicity of *S. hortensis* L. oil showed that the oil from this plant and its main component—carvacrol (which accounts for about 80% of volatile compounds in the F2 vegetation phase when polyphenols rutin and rosmarinic acid content was highest) was highly cytotoxic for human breast cancer cells—MDA MB231 and therefore it has been suggested that this compound may have potential therapeutic significance in the treatment of cancer [42]. However, so far, the cytotoxicity of this monoterpene for epidermal cells (both normal and neoplastic) has not yet been analyzed. The obtained results indicate that F2 extract from *S. hortensis* L. may have a differentiating effect on the response of normal and cancer cells because this extract is cytotoxic to melanoma already at a concentration of 12.5 μg/mL, in the absence of cytotoxicity to melanocytes. Therefore, this extract may be a potential therapeutic element in relation to skin melanoma.

Regardless of the effect of the *S. hortensis* L. extract on cell survival, its antioxidant activity has been demonstrated, associated with the polyphenols contained in this extract. The antioxidant properties of *S. hortensis* L. extracts have been directly assessed, one of the most used methods for assessing the antioxidant activity of natural oils/extracts with the ability to scavenge DPPH radicals. It has been shown that radicals most effectively capture antioxidants contained in *S. hortensis* L. extract from the V2 phase of vegetation. At the same time, in this phase of vegetation, *S. hortensis* L. contains the largest number of polyphenols, including flavonoids. Literature data confirm that there is a direct relationship between the content of polyphenolic compounds and the antioxidant activity of most plant extracts/components [43]. The antioxidant activity of polyphenols is associated with hydroxyl groups associated with the aromatic ring, which are able donate hydrogen atoms with electrons and stabilize free radicals [44]. The antioxidant activity of these compounds is also associated with the ability to chelate transition metal ions and quench singlet oxygen. The consequence of polyphenol antioxidant activity is also their participation in lipid stabilization by preventing their peroxidation [45,46]. Therefore, due to the different mechanisms of possible antioxidant activity, polyphenols can effectively inhibit phospholipid metabolism particularly lipid peroxidation that occurs on enzymatic and non-enzymatic ways following cells exposure to UV radiation contained e.g., in sunlight. Despite of the multiple pathways of UV radiation action, dependent to a large extent on its wavelength, exposure to it usually leads to the intracellular pro-oxidative pathways induction and oxidative stress occurring, which results in the lipid oxidation upon lipid radical formation [47]. Oxidative chain reactions are initiated by non-enzymatic lipid peroxidation initiators such as hydroxyl and hydroperoxyl radicals. Membrane phospholipids containing polyunsaturated fatty acids (PUFAs), namely arachidonic, linoleic, linolenic, eicosapentaenoic, and docosahexaenoic acids mostly suffer from UV-induced peroxidation.

Due to the action of reactive oxygen species on membrane phospholipids, fragmentation produces unsaturated short chain, reactive aldehydes, such as MDA, while cyclic prostaglandin derivatives result from oxidative cyclization [48]. In addition, endocannabinoids and eicosanoids are generated as a result of enzymatic metabolism with the participation of cyclooxygenase and lipoxygenase (COXs/LOXs) [49]. The production of these biologically active compounds can disrupt cellular metabolism, but above all, through the metabolism of membrane PUFAs, it affects the modification of the structure and function of biological membranes. Therefore, most studies on the effects of polyphenolic antioxidants focus on their effects on biological membranes, including preventing changes in their structure as a result of the peroxidation process. The results of this work show that the higher level of polyphenols in the *S. hortensis* L. extract corresponds to the reduction in the level of lipid peroxidation product, which is MDA. Extracts from the vegetative phases F1-3 characterized by a lower level of all phenolic compounds also had a higher level of MDA compared to control cells. In the case of treating cells with extracts from the V1 and V2 phases with low concentrations, a significantly higher level of phenolic compounds was accompanied by preventing the increase in MDA levels even after UV radiation.

Physical properties and functionality of cell membranes are directly affected by formation of lipid peroxidation products. The lipid asymmetry is interrupted reducing the hydrophobicity of the inner layer of lipid membrane causing depolarization [50]. As a result, membrane integrity may be lost [51]. Regardless of the above, lipid peroxidation products are chemically reactive molecules due to their carbon-carbon double bond structure and electrophilic characteristics, which reacts with the cell’s nucleophilic components, including DNA, lipids, peptides, and proteins, leading to disturbances in cell metabolism. During these reactions, free reactive lipid peroxidation products decrease, and at the same time adducts with proteins increase that promote disruption of cell signaling and affect metabolic modifications resulting in cell dysfunction and apoptosis [52].

Regardless of the antioxidant activity of the polyphenols contained in *S. hortensis* L. extracts, other components of the extract can completely modify the lipid peroxidation process. Earlier it was pointed out that carvacrol present in very high concentration in *S. hortensis* L. extract may increase the permeability of bacterial cell membranes, which leads to their death [53]. It can be suggested that the results of this study confirm the opposite effect of carvacrol on lipid peroxidation than polyphenols. The consequence of this is an increase in lipid peroxidation for extracts from V2 phase, full flowering and end of flowering, and at higher concentrations of extracts, and therefore even higher carvacrol concentration.

Moreover, the higher lipid peroxidation after using extracts V1 and V2 may also be caused by lowest level of rutin and rosmarinic acid in these extracts. Both these polyphenols are powerful antioxidants, and their higher level in F1–F3 extracts guarantees protection of membrane phospholipids. Rutin that is known for its antioxidant activity, in the case of other skin cells, has been shown as a molecule that not only directly reacts with free radicals but also inactivates enzymes responsible for their generation [28]. Furthermore, rutin protective effect on the skin relates to its involvement in intracellular signaling leading to cytoprotective proteins expression, antioxidant enzymes activation, as well as apoptosis prevention [54]. Moreover, rutin antioxidant action significantly prevents lipid oxidative metabolism, which might be observed as lower lipid peroxidation products level, but also decreased level of other lipid metabolism markers such as isoprostanes or endocannabinoids, which are known as pro-inflammatory mediators [55]. On the other hand, rutin also shows cytotoxic action against various types cancer cells [56,57,58], which can be connected with a higher level of lipid peroxidation products in cancer cell compared to non-transformed cells what was also observed in this study. Simultaneously, also rosmarinic acid that level is the highest in studied extracts, additionally increases skin cells antioxidant capacity. It has also been shown that rosmarinic acid can stimulate the activity of antioxidant enzymes such as glutathione peroxidase, catalase, and superoxide dysmutase [59,60], as well as prevent lipid peroxidation [61]. The cytoprotective action is visible mainly in skin melanocytes, but not in the case of melanoma, which is partially confirmed by data for other cancer types [62].

## 5. Conclusions

This manuscript results have been shown that the V2 phase alcohol extract of *S. hortensis* L. contains a high amounts of polyphenols, including rutin and rosmarinic acid, which promotes the radical scavenging activity and antioxidant properties of this extract. In contrast, the extract from the F2 phase already at a concentration of 12.5 µg/mL reduces the survival of melanoma cells to 60% with comparison to 90% for melanocytes. In addition, extracts from the beginning of flowering and end of flowering at a concentration of 25 µg/mL, containing significantly less rutin and rosmarinic acid, in combination with irradiation of cells with UVB, significantly increased the lipid peroxidation process, particularly in melanoma cells. These data indicate the possibility of using extracts from *S. hortensis* L to modulate/differentiate the metabolism of normal and tumor skin cells.

## Data Availability

The primary data summarized in this study are available on request from the corresponding author. The raw data are not publicly available due to low value when not analyzed, compared and summarized.
